# Microbiological assessment of silver nanoparticles versus minocycline

**DOI:** 10.6026/973206300210836

**Published:** 2025-04-30

**Authors:** Nalini M.S, Arvind Raghunath, Sravani Bontu, Surya Sreevatsa C.M, Pranita H.

**Affiliations:** 1 Department of Periodontology, Raja Rajeswari Dental College and Hospital, Bangalore, Karnataka, India

**Keywords:** Local drug delivery, silver nanoparticles gel, minocycline gel, chronic periodontitis

## Abstract

Evaluation of 0.02% silver nanoparticle (AGNP) gel and 2% minocycline gel as adjuncts to scaling and roots planning (SRP) in chronic periodontitis is of interest. Hence, 30 patients with 90 sites were divided into three groups: SRP alone, SRP + AGNP and SRP + minocycline. Clinical parameters improved significantly in all groups at 3 months (p < 0.001). AGNP and minocycline showed superior outcomes to SRP alone, with no significant difference between them. Thus, AGNP gel is as effective as minocycline and may serve as a promising adjunctive therapy.

## Background:

Periodontal diseases are classified as infections of the periodontium due to their bacterial origin, immune response involvement and resulting tissue destruction [1]. The clinical signs of periodontitis are changes in the morphology of gingival tissues, bleeding upon probing as well as periodontal pocket formation. This pocket provides an ideal environment for the growth and proliferation of anaerobic pathogenic bacteria [2]. The microflora associated with periodontitis is complex and primarily consists of Gram-negative anaerobic bacteria [3]. Therapeutic approaches, such as mechanical scaling, root planning and occasionally surgery, help reduce gingival inflammation and clinical probing depth due to treatment [4]. Systemic antimicrobials have been recommended for treating severe periodontitis, but they can cause side effects such as hypersensitivity, gastrointestinal issues and the development of bacterial resistance [5, 6]. The local concentration of a drug within tissues can be increased by embedding the active agent into controlled-release delivery systems, which are then placed directly into the periodontal pocket. *In vitro* studies have demonstrated that minocycline hydrochloride is highly effective against most microorganisms involved in periodontal disease. Additionally, among all tetracyclines, minocycline exhibits the highest substantivity and superior lipid solubility [7]. Numerous studies have examined the clinical and microbiological impacts of this gel in both beagles and humans. Following scaling and root planning, Periocline was applied weekly for four consecutive weeks. By the end of week four, treated sites showed a significant reduction in the levels of *Porphyromonas gingivalis* and *Actinobacillus actinomycetemcomitans*. At week four, a *Prevotella intermedium* was present in seven out of 22 sites, increasing to 16 sites by week 12 in the treated areas. Furthermore, sites treated with minocycline exhibited a significant decrease in probing depth and bleeding on probing compared to control sites at week four [8]. Recent advancements in nanotechnology have led to the development of innovative therapeutic materials for treating periodontal diseases. Nanoparticles, which are clusters of atoms, generally range in size from 1 to 100 nm [9]. Silver nanoparticles attach to the bacterial cell wall, enabling their penetration and inducing structural modifications that alter cell permeability, ultimately resulting in cell death. The antimicrobial efficacy of a silver nanoparticle gel (0.02 mg/g) has been observed at 3.125 µg/ml against *P. gingivalis* and 6.25 µg/ml against *A. actinomycetemcomitans*, making it a promising option for applications in nanomedicine [10]. Therefore, it is of interest to assess the effectiveness of subgingival local drug delivery of silver nanoparticle gel compared to minocycline gel in patients with chronic periodontitis.

## Materials and Methods:

This randomised, split-mouth, double-blind clinical trial was conducted in the Department of Periodontology. Thirty patients, male and female, aged between 25 and 60 years and diagnosed with chronic periodontitis, were enrolled. A total of 90 sites were randomly selected from the outpatient section for the study. The inclusion criteria were as follows: patients with probing pocket depths of 4-8 mm, no history of periodontal therapy in the previous six months, systemic health, good oral hygiene and willingness to comply with follow-up visits. Exclusion criteria included pregnant or lactating women, individuals allergic to minocycline or silver nanoparticles, smokers and those who had taken medications affecting periodontal status within the last six months. The study's purpose and design were fully explained to the participants and written consent was obtained from each patient. The selected sites in each patient were randomly assigned to one of three groups using the coin flip method. Group 1 underwent scaling and root planning followed by the subgingival delivery of 0.02% silver nanoparticle gel. Group 2 underwent scaling and root planing followed by the subgingival delivery of 2% minocycline gel. Group 3 underwent scaling and root planing alone. Potential participants were screened using a mouth mirror and a UNC-15 periodontal probe to evaluate their periodontal status. Chronic periodontitis was diagnosed according to the criteria established during the 1999 International Workshop for the Classification of Periodontal Diseases. Silver nanoparticle gel was formulated by combining hydroxypropyl methylcellulose powder and silver nanoparticle powder with distilled water, followed by continuous stirring. Minocycline gel was prepared by incorporating 2% minocycline hydrochloride (HCl) into a matrix composed of hydroxyethyl cellulose, aminoalkyl methacrylate, triacetin and glycerin, with magnesium chloride added to modulate drug release. Site selection was based on probing pocket depths, ensuring a minimum separation of two teeth between treatment modalities when the test and control sites were located in the same jaw. At the baseline visit, probing pocket depth (PPD) and clinical attachment level (CAL) were measured. Periodontal therapy consisted of full-mouth scaling and root planing, along with oral hygiene instructions. Patients were monitored and those presenting with persistent periodontal pockets (PPD of 4-6 mm) were selected for further evaluation. Alginate impressions were taken to fabricate acrylic occlusal stents, ensuring standardized clinical recordings throughout the study. One month later, the acrylic occlusal stents were placed at the test sites, baseline measurements were recorded and silver nanoparticle gel and minocycline gel were injected into the pockets. A periodontal dressing was applied over the treated areas and patients returned after seven days for the removal of the dressing. Patients in Group 3 underwent scaling and root planing only, with their recall schedule aligned with Groups 1 and 2. Throughout the study, patients were instructed to avoid chemical plaque control methods and were recalled for follow-up evaluations at one and three months post-treatment. Clinical parameters, including the Plaque Index (PI), Gingival Index (GI), Probing Pocket Depth (PPD) and Clinical Attachment Level (CAL), were recorded at baseline, one month and three months. The PPD and CAL were measured using a UNC-15 periodontal probe, with all measurements performed by a single examiner. Subgingival plaque samples were collected from the test sites at baseline and cultured to determine the colony-forming units (CFUs) of anaerobic bacteria. This procedure was repeated at three months. Statistical analysis was conducted using SPSS for Windows, Version 22.0. Descriptive statistics were calculated for quantitative and categorical variables, including means, standard deviations and proportions. One-way ANOVA followed by Tukey's post hoc analysis was performed to compare clinical parameters and mean CFUs across groups at different time intervals. Repeated measures ANOVA with Bonferroni's post hoc test was used to evaluate mean PI, GI, PPD and CAL across time intervals within each group. The Kruskal-Wallis test was employed to compare CFUs between groups at baseline and at three months, while the Wilcoxon signed-rank test was used to compare means within each group over time. A significance level of P < 0.05 was considered statistically significant.

## Results:

The study was conducted October 2021 to November 2022. A total of 30 patients contributing to 90 sites entered the study at baseline. All the patients tolerated the locally delivered 0.02% Silver Nanoparticles gel & 2% Minocycline gel used in the study well & did not report any adverse reactions. The mean plaque index (PI) score in the inter-group comparison for Group 1 at baseline was 1.87 ± 0.23, which reduced to 0.97 ± 0.21 at 1 month and increased marginally by 1.25 ± 0.16 at 3 months. A statistically significant difference was noted between Groups 1 & 3 and between Groups 2 & 3, at 1 month and 3 months. There was no statistical difference between Groups 1 & 2 at all-time intervals (Table 1). In the intra-group comparison for PI, there was a statistically significant reduction in all the 3 groups at different time intervals [BL, 1Month, 3Month, p-value <0.001)] The mean Gingival index(GI) score for Group 1 at baseline was 1.73 ± 0.40, which reduced to 0.69 ± 0.20 and marginally increased by 1.00 ± 0.23 at 3 months. There was a statistically significant difference between Group 1 & Group 3 (0.69 ± 0.20 & 1.03 ± 0.17 respectively, p-value <0.001) and between Group 2 & Group 3 (0.79 ± 0.22 & 1.03 ± 0.17 respectively, p-value <0.001) at 1 month and statistically significant differences were noted between Group 1 & Group 3 (1.00 ± 0.23 & 1.25 ± 0.26 respectively, p-value <0.001) at 3 months. There was no statistical difference between Group 1 & Group 2 at all-time intervals (Table 2). In intra-group comparison for GI, there was a statistically significant reduction in all the 3 groups at all-time intervals [BL, 1Month, 3Month, p-value <0.001]. The mean pocket depth (PD) for Group 1 at baseline was 5.57 ± 0.50 which reduced to 4.53 ± 0.57 at 1 month and 2.73 ± 0.45 at 3 months. At 3 months there was a statistical difference between Group 1 & Group 3 (2.73 ± 0.45 & 3.63 ± 0.49 respectively, p<0.001) and between Group 2 & Group 3 (2.80 ± 0.61 & 3.63 ± 0.49 respectively, p<0.001). In the intra-group comparison for PD, there was a statistically significant reduction in PD in all 3 groups at all-time intervals (BL, 1Month, 3Month, p<0.001). The mean Clinical attachment level(CAL) for Group 1 at baseline was 6.47 ± 1.11 which reduced to 5.43 ± 1.17 at 1 Month and 3.37 ± 1.19 at 3 Months. At 3 months there was a statistically significant difference between Group 1 & Group 3 (3.37 ± 1.19 & 4.57 ± 1.10, respectively, p-value<0.001) and between Group 2 & Group 3 (3.90 ± 1.06 & 4.57 ± 1.10, respectively, p-value 0.01). In intra-group comparison, there was a significant reduction in CAL among all groups at all-time intervals (BL, 1Month, 3Month, p-value<0.001). The mean Colony forming units (CFU) CFUs/ml for Group 1 at baseline was 5.00 ± 0.45 which reduced to 1.93 ± 0.58 at 3 months and in Group 2 the mean CFUs/ml at baseline was 4.87 ± 0.51 which reduced to 2.10 ± 0.61 at 3 months. At 3 months, there was a statistically significant difference between Group 1 & Group 3 (1.93 ± 0.58 & 2.83 ± 0.59, respectively; p-value<0.001) and between Group 2 & Group 3 (2.10 ± 0.61 & 2.83 ± 0.59, respectively; p-value<0.001). In intra-group comparison, there was a significant decrease in CFUs/ml count in all groups at all-time intervals (BL, 3Months, p-value<0.001).

## Discussion:

This split-mouth randomized clinical trial aimed to evaluate and compare the efficacy of 0.02% silver nanoparticles and 2% minocycline gel as adjuncts to scaling and root planning in the treatment of chronic periodontitis. Clinical parameters, including the plaque index (PI), gingival index (GI), probing pocket depth (PPD) and clinical attachment level (CAL) were recorded at baseline, 1 month and 3 months. Additionally, colony-forming units (CFUs) were assessed at baseline and 3 months post-therapy. A study by Steckiewicz *et al.* (2022) evaluated the effectiveness of silver nanoparticles (AgNPs) combined with chlorhexidine (AgNPs-CHL) or metronidazole (AgNPs-PEG-MET) in the treatment of periodontitis. The study found a significant improvement in the plaque index (PI) [11]. In this study, the Gingival Index (GI) showed a significant reduction in all three groups from baseline to one month, with a further decrease observed at three months. When compare the groups, both Group 1 and Group 2 demonstrated superior outcomes, with a p-value of <0.001. However, no statistically significant difference was observed between the two groups. These findings align with the study conducted by Kale *et al.* which evaluated the effectiveness of silver nanoparticle gel in patients with chronic periodontitis and reported a significant reduction in the GI from baseline to three months [10]. In a split-mouth clinical trial by Lu *et al.* (2005), the addition of subgingival minocycline to scaling and root planning led to a statistically significant decrease in GI at 10, 14 and 18 weeks [12]. The probing pocket depth (PPD) in this study demonstrated a statistically significant reduction at all-time points across all groups. In the intra-group comparison, both Group 1 and Group 2 (test groups) showed a significant decrease from baseline to 3 months, with a p-value of less than 0.001, whereas Group 3 (control group) did not show similar results. However, there was no significant difference observed between Group 1 and Group 2. A study by Paquette *et al.* (2002) reported comparable results, with significant reductions in probing depth (PD) at 1, 3, 6 and 9 months following the assessment of minocycline microspheres [13. The results align with the findings of Kale *et al.*, who demonstrated that the group treated with silver nanoparticles showed a significant decrease in periodontal depth from baseline to both 1 month and 3 months (p-value < 0.05) [10]. There was a significant improvement in the clinical attachment level in all three groups from baseline to 3 months. However, when comparing between groups, a statistically significant difference was observed between Groups 1 and 2, as compared to Group 3, at 3 months, with a p-value of < 0.01. No significant difference was found between Group 1 and Group 2.

In a study involving 2% minocycline gel, a significant reduction in attachment level improvement was observed in comparison to the control group [14]. In our study, both Group 1 (0.02% silver nanoparticles gel) and Group 2 (2% minocycline gel) exhibited significant decreases in probing depth (PD) and clinical attachment level (CAL), likely due to their well-known anti-inflammatory properties. Minocycline offers additional advantages as a strong anti-inflammatory agent. Research indicates that it inhibits the activity of collagenolytic enzymes produced by host tissues during inflammation by binding to Ca++ and Zn++. Furthermore, Golub, Gabler and Creamer showed that minocycline application suppresses various neutrophil functions, such as migration, O2 synthesis and degranulation, all of which contribute to tissue destruction during inflammation [15, 16]. In a study conducted by Nadworny *et al.* there was a noticeable reduction in both visual and histological markers of inflammation, along with a decrease in the expression of gelatinases and pro-inflammatory cytokines, including transforming growth factor (TGF)-β, tumour necrosis factor (TNF)-α and interleukin (IL)-8 [17]. The study evaluated the effectiveness of silver nanoparticle gel and minocycline gel in combating anaerobic microorganisms. The microbiological analysis showed a statistically significant decrease in colony-forming units (CFUs) across all three groups when comparing baseline data with the data collected at the 3-month mark. Silver nanoparticles derived from Ocimum sanctum demonstrated improved antimicrobial activity against Fusobacterium nucleatum, *Porphyromonas gingivalis*, Aggregatibacter actinomycetemcomitans and Prevotella intermedia [18]. Silver nanoparticles can accumulate in the cell wall pits after binding to the cell surface [19]. Accumulated nanoparticles can cause damage to the cell membrane. Due to their minuscule size, silver nanoparticles can penetrate bacterial cell walls and alter the structure of the cell membrane. This alteration may lead to the rupture of organelles and, in some cases, cell death. Furthermore, silver nanoparticles can disrupt bacterial signal transmission by interfering with protein phosphorylation. They can remove phosphate groups from tyrosine residues on proteins, thereby disturbing signal transmission, potentially resulting in cell death and halting cell division [20]. Gram-negative bacteria are more susceptible to silver nanoparticles because their cell walls are thinner compared to those of gram-positive bacteria. The thicker cell walls of gram-positive bacteria can make it more difficult for nanoparticles to penetrate their cells [21]. A study by Umeda *et al.* (1996) investigated the effects of minocycline gel on bacteria in periodontal pockets. Following the application of the gel, there was a significant reduction in the numbers of *Porphyromonas gingivalis* and *Tannerella forsythia* [22]. In a 2017 study by Soeroso *et al.* Minocycline HCL 2% gel was used in conjunction with scaling and root planing (SRP) to treat chronic periodontitis. This treatment resulted in a significant decrease in the bacteria *Porphyromonas gingivalis*, *Tannerella forsythia* and Treponema denticola over a six-month period [23]. In this study, both 0.02% silver nanoparticles gel and 2% minocycline gel were found to be both clinically and microbiologically effective when used alongside scaling and root planning to treat chronic periodontitis. No complications were reported in patients during the study. However, the limitations of this study could stem from the short follow-up period. Future studies with extended follow-up durations are required to assess the long-term effects of both gels in patients with chronic periodontitis.

## Conclusion:

All study participants showed a significant decrease in the mean plaque index (PI), gingival index (GI), probing pocket depth (PPD), clinical attachment level (CAL) and colony forming units (CFUs). Over 3 months, noticeable improvements in clinical parameters were seen after scaling and root planning, along with the application of 0.02% silver nanoparticle gel under the gums. The results showed that the improvements from the silver nanoparticles gel were similar to those achieved with 2% minocycline gel. These findings suggest that 0.02% silver nanoparticles gel is an effective addition to scaling and root planing in treating chronic periodontitis, with similar benefits to 2% minocycline gel.

## Source of funding:

None

## Figures and Tables

**Table 1:** Comparison of mean Plaque Index scores b/w groups at different time intervals using Kruskal Wallis Test followed by Dunn's Post hoc Test

**Time**	**Groups**	**N**	**Mean**	**SD**	**p-value^a^**	**Sig. Diff**	**p-value^b^**
Baseline	Group 1	30	1.87	0.23	0.67	G1 vs G2	..
	Group 2	30	1.88	0.34		G1 vs G3	..
	Group 3	30	1.93	0.27		G2 vs G3	..
1 Month	Group 1	30	0.97	0.21	0.01*	G1 vs G2	0.39
	Group 2	30	1.03	0.18		G1 vs G3	0.01*
	Group 3	30	1.1	0.1		G2 vs G3	0.09
3 Months	Group 1	30	1.25	0.16	0.002*	G1 vs G2	0.77
	Group 2	30	1.22	0.16		G1 vs G3	0.02*
	Group 3	30	1.36	0.12		G2 vs G3	0.002*
* - Statistically Significant
Note: a. Kruskal Wallis Test & b. Dunn's Post hoc Test

**Table 2:** Comparison of mean Gingival Index scores b/w groups at different time intervals using Kruskal Wallis Test followed by Dunn's Post hoc Test

**Time**	**Groups**	**N**	**Mean**	**SD**	**p-value^a^**	**Sig. Diff**	**p-value^b^**
Baseline	Group 1	30	1.73	0.4	0.83	G1 vs G2	..
	Group 2	30	1.71	0.41		G1 vs G3	..
	Group 3	30	1.77	0.32		G2 vs G3	..
1 Month	Group 1	30	0.69	0.2	<0.001*	G1 vs G2	0.14
	Group 2	30	0.79	0.22		G1 vs G3	<0.001*
	Group 3	30	1.03	0.17		G2 vs G3	<0.001*
3 Months	Group 1	30	1	0.23	<0.001*	G1 vs G2	0.81
	Group 2	30	0.97	0.19		G1 vs G3	<0.001*
	Group 3	30	1.25	0.26		G2 vs G3	<0.001*
* - Statistically Significant
Note: a. Kruskal Wallis Test & b. Dunn's Post hoc Test
